# Deep multiscale feature fusion network with dual attention for rolling bearing remaining useful life prediction

**DOI:** 10.1038/s41598-025-97380-x

**Published:** 2025-04-28

**Authors:** Yingming Yang, Zhihai Wang, Xiaoqin Liu, Tao Liu, Zhuopeng Luo

**Affiliations:** 1 Faculty of Mechanical and Electrical Engineering, Kunming University of Science and Technology, Kunming, 650500 China; 2 Key Laboratory of Advanced Equipment Intelligent Manufacturing Technology of Yunnan Province, Kunming University of Science and Technology, Kunming, 650500 China

**Keywords:** RUL prediction, Rolling bearing, Dual attention mechanism, Multi-scale residual pyramid, Transformer, Relative position coding, Engineering, Mechanical engineering

## Abstract

Aiming at the existing life prediction methods for rolling bearing degradation information mining is not sufficient, the critical time step information degree is insufficient, resulting in the loss of key degradation information, model prediction accuracy and model generalization ability is insufficient, this paper proposes a novel deep multiscale feature fusion network with dual attention for rolling bearing remaining useful life (RUL) prediction. First, multi-domain feature sets of rolling bearing vibration signals are acquired. Subsequently, it is proposed to use Squeeze-and-Excitation (SE) attention mechanism to calibrate and weigh temporal significance of feature sequences, thereby capturing critical temporal information. Then, a multi-scale feature extraction and fusion module with deep network is constructed, consisting of multiple identical multi-scale residual pyramid layers connected in series to further delve into the state information of rolling bearings. Additionally, a relative position encoding method suitable for time series prediction is introduced within the multi-head attention mechanism, a network architecture based on a dual attention-enhanced Transformer encoding layer is established. This enhancement significantly improves model prediction accuracy, generalization capability, and sequence data comprehension ability. Finally, the high-level features output from the feed-forward layer are mapped through a regression layer to obtain the final prediction results. Experimental results demonstrate the superior performance of the proposed method in terms of both prediction accuracy and generalization capability for rolling bearing life prediction. A more robust and accurate framework for RUL prediction in rolling bearings is provided.

## Introduction

As a key component of rotating mechanical equipment, rolling bearings have a direct impact on the performance, reliability and safety of the equipment. Therefore, carrying out the remaining life prediction of rolling bearings is of great engineering significance and practical necessity for preventing equipment failures, formulating maintenance strategies and guaranteeing production safety. At present, the life prediction of rolling bearings mainly uses two methods: the modeling method and the data-driven method^1^. The model approach uses mathematical and physical knowledge to model the degradation process, but accurate modeling of complex systems is often very difficult^2^. With the development of deep learning, data-driven methods have been widely used in rolling bearing life prediction, which requires less or even no in-depth knowledge of specific domain expertise to mine bearing degradation information and establish RUL mapping relationships^3^.

To obtain the rolling bearing fatigue key information, the state signal needs to be feature extracted to obtain the fatigue-sensitive indicator set, and at the same time reduce the model training efficiency. Currently, the mainstream feature extraction is mainly multi-domain extraction such as time domain, frequency domain, etc. and feature extraction from the original signal by deep learning network^4–7^. Deep network-based feature extraction relies on a large amount of labeled data^8,9^ and it is difficult to obtain data and time-consuming to fine-tune the network in real industrial scenarios. In contrast, direct traditional feature extraction in time and frequency domains has low data and hardware requirements and is more suitable for rapid deployment and application.

In the past years, the Transformer coding layer based on a multi-head self-attention mechanism has become a research hotspot in life prediction schemes^10–12^ due to its ability to capture long-range dependencies of sequences and computationally efficient factors such as Zhang captured performance degradation patterns by bearing operation vibration and temperature data via Transformer architecture, which improves the prediction accuracy^13^. Wang introduced a rolling bearing life prediction method combining a Transformer encoder and multi-task learning, which outperforms the traditional method^14^. Li proposed a hybrid deep learning model combining the attention mechanism to predict the bearing life, which shows better performance^15^. The above prediction methods are effective though. However, in actual operation, the working conditions and failure modes of bearings are complex and variable, and their states often show a dynamic transition between long-term and short-term, and the monitoring data contain a large number of long- and short-term features. Most of the existing models, including Transformer, are limited to global or local information extraction, which usually focuses on a single internal scale, i.e., the sensory field feature extraction is unable to capture the degradation information at multiple time scales, resulting in inadequate characterization of the degradation information. Although scholars have tried to connect the convolutional layer after the Transformer normalization layer to alleviate the problem^16^, only limited local information extraction can be obtained, and the lack of information can still not be avoided. The quality of input data still needs to be further strengthened, and a strategy that can highlight the critical time-step information in the whole chain of feature extraction and temporal modeling needs to be proposed urgently.

In summary, to solve the above problems of insufficient mining of degradation information, loss of key degradation information due to insufficient critical time step information, insufficient model prediction accuracy and insufficient model generalization ability, this paper proposes a method for predicting the remaining service life of rolling bearing based on the multi-scale feature fusion of the deep network and the enhancement of the dual-attention mechanism. The critical degradation time step information is calibrated by the SE attention mechanism to enhance the rolling bearing fatigue data quality. The tandem multi-scale residual pyramid layer is constructed to deeply mine the bearing degradation information to further enhance the model degradation characterization capability. Meanwhile, relative position-coding^17^ is introduced in the multi-head attention module to strengthen the temporal dependency between time steps and thus capture key information.

The main framework of this paper is as follows. Section “Transformer structure and its attention mechanism” introduces the important components of the Transformer coding layer, residual attention, and the principle and structure of the feature pyramid, Section “Proposed method” describes the data preprocessing, the theory of the proposed modeling method and the prediction process in this paper, and Section “Experimental verification” focuses on the model performance metrics, discussion of the main layers’ hyper-parameters, experimental validation of the model, and the experimental ablation of the relative position coding. Section “Analysis and discussion” verifies the model’s generalizability. Finally, Section “Conclusion” summarizes the paper.

## Transformer structure and its attention mechanism

**Fig. 1:**
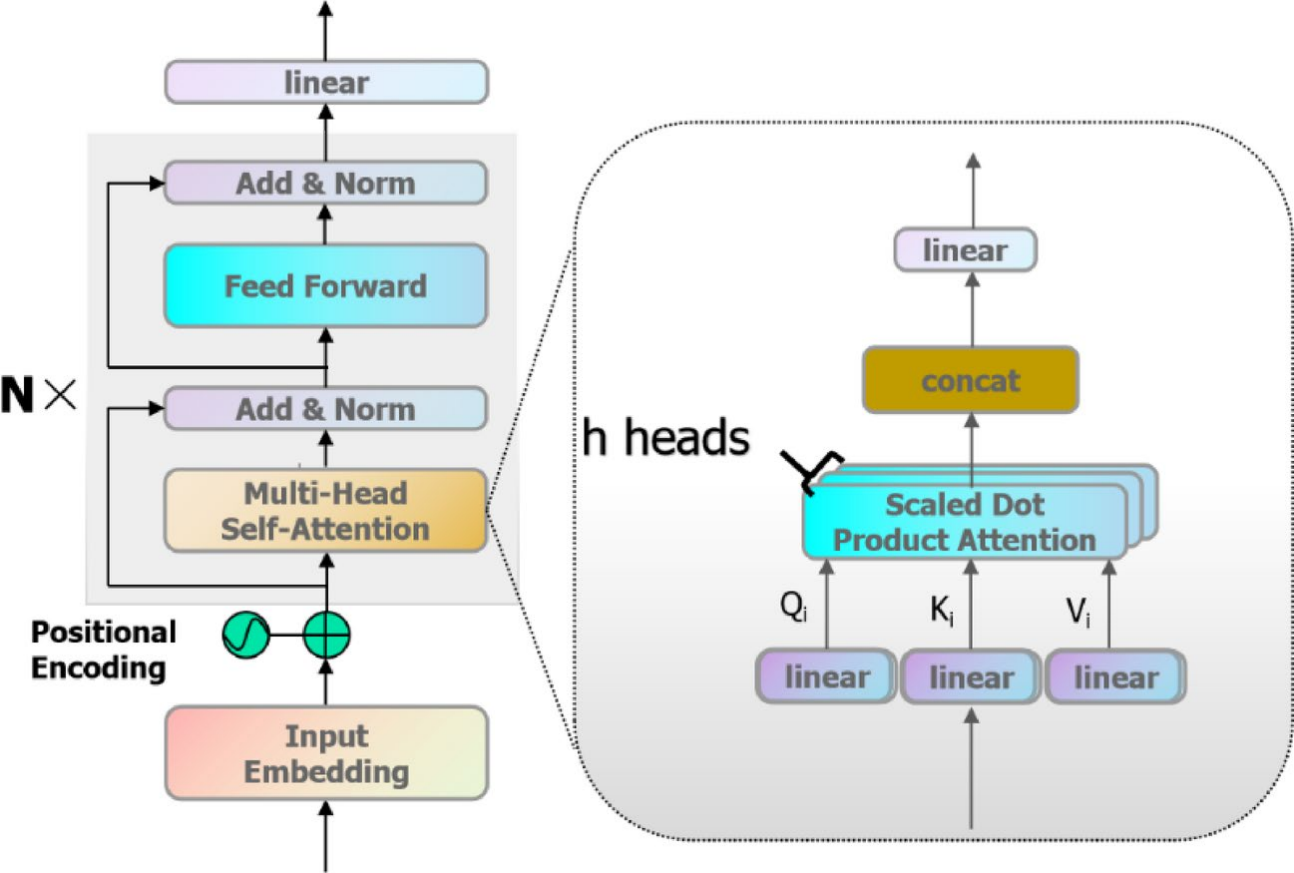
Transformer encoder layer structure.

A transformer network with its self-attention mechanism can effectively capture the long-range dependencies between data sequences, and its parallel computing characteristics, which can significantly improve the efficiency of model training, is particularly suitable for dealing with such long-time sequence data as the full-life signals of rolling bearings. Given the complexity of the decoder structure and the dependence on the output of the previous sequence that may lead to an increase in computational cost and memory consumption^18^, this paper chooses to carry out the study of life prediction by using the encoder structure of Transformer, as shown in Fig. 1. The encoding layer consists of multi-head self-attention, layer normalization and feed-forward layers, and the effect of network depth on gradient training is mitigated by residual connectivity.

### Multiple attention mechanisms

The multi-head attention mechanism extends self-attention with multiple parallel attention heads, each of which independently computes the attention value of the feature matrix. Multi-head attention can focus on features in different subspaces simultaneously stitch this information and transform it linearly to obtain the final output, improving computational efficiency. See Fig. 1 and Eq. (1)

1 $$\begin{gathered} Q_{i} = XW_{i}^{q} \hfill \\ K_{i} = XW_{i}^{k} \hfill \\ V_{i} = XW_{i}^{v} \hfill \\ d_{h} = \frac{{d_{{{\text{model}}}} }}{n} \hfill \\ h_{i} = Attention\left( {Q,K,V} \right)_{{\text{i}}} = {\text{softmax}} \left( {\frac{{Q_{i} K_{i}^{T} }}{{\sqrt {d_{h} } }}} \right)V_{i} \hfill \\ MultiHead\left( {Q,K,V} \right) = Concat\left( {h_{1} ,h_{2} ,...,h_{n} } \right)W \hfill \\ \end{gathered}$$

where: $X$ represents the local input data of the model, $n$ represents the total number of attention heads $W_{i}^{q} ,W_{i}^{k} ,W_{i}^{v}$ represent the query matrix $Q$, keys matrix $K$ and values matrix $V$ trainable weight matrix under the $ith$ head of attention, respectively, $d_{h}$ is the embedding dimension $d_{{{\text{model}}}}$ divided by the number of attention heads $n$, denoting the dimension of a single head to prevent gradient explosion, $h_{i}$ represents the value of the attention under the $ith$ head of attention, and $W$ represents the trainable weights matrix after combining the multiple heads of attention.

### Residual attention mechanism

**Fig. 2:**
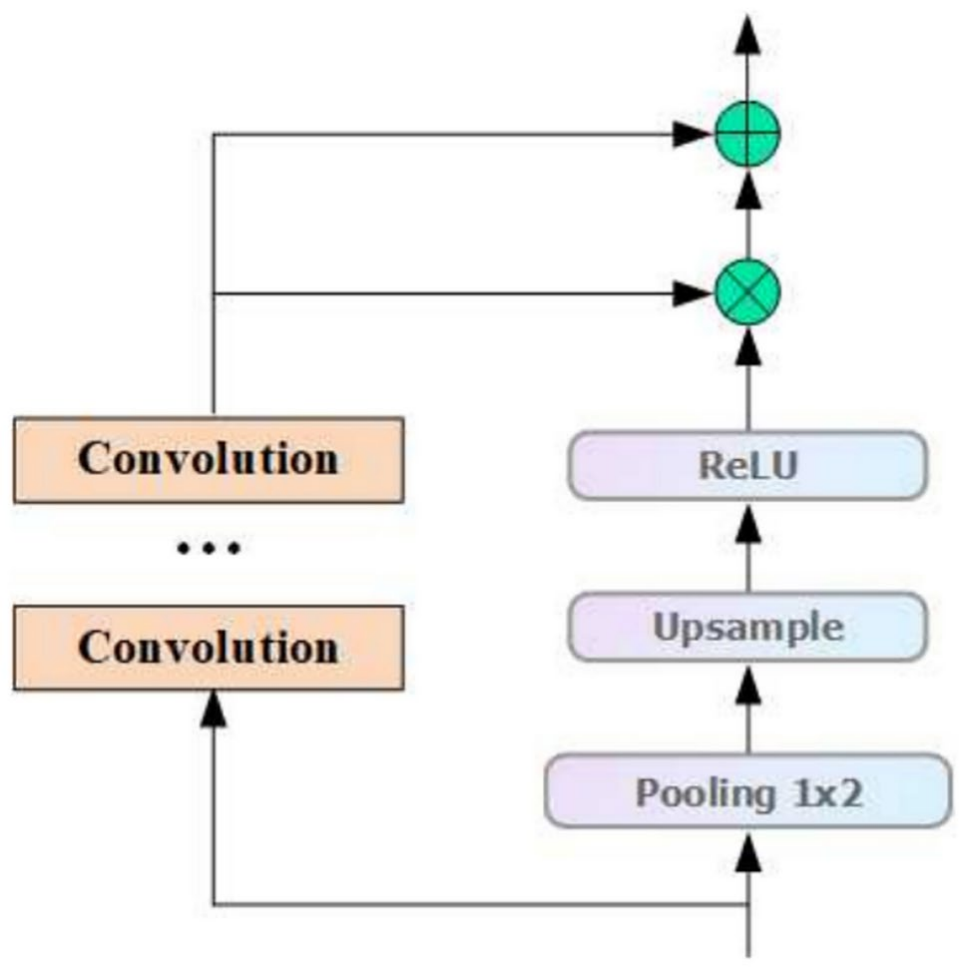
Structure of residual attention.

Residual attention^19–21^ was originally proposed by He et al. in ResNet to overcome the difficulties in deep network training. In this paper, we propose to combine residual attention with the feature pyramid attention mechanism to construct an innovative feature extraction framework. The framework consists of a backbone branch that performs a convolutional operation to extract features and adjusts the structure according to the demand, and a mask branch that generates a mask of the same size as the output of the backbone through the up-sampling and down-sampling attention mechanism, which serves as a weight matrix for local optimization of the features. The structure of the residual attention layer and its calculation formula are detailed in Fig. 2 and Eq. (2).

2 $$H(x) = (1 + M(x^{\prime})) \times V(x^{\prime})$$

where: $x^{\prime}$ is the input feature data, $M(x^{\prime})$ is the residual attention layer mask branch output features, and $V(x^{\prime})$ is the convolution layer of the main branch Convolution.

### Feature pyramid attention mechanism

**Fig. 3:**
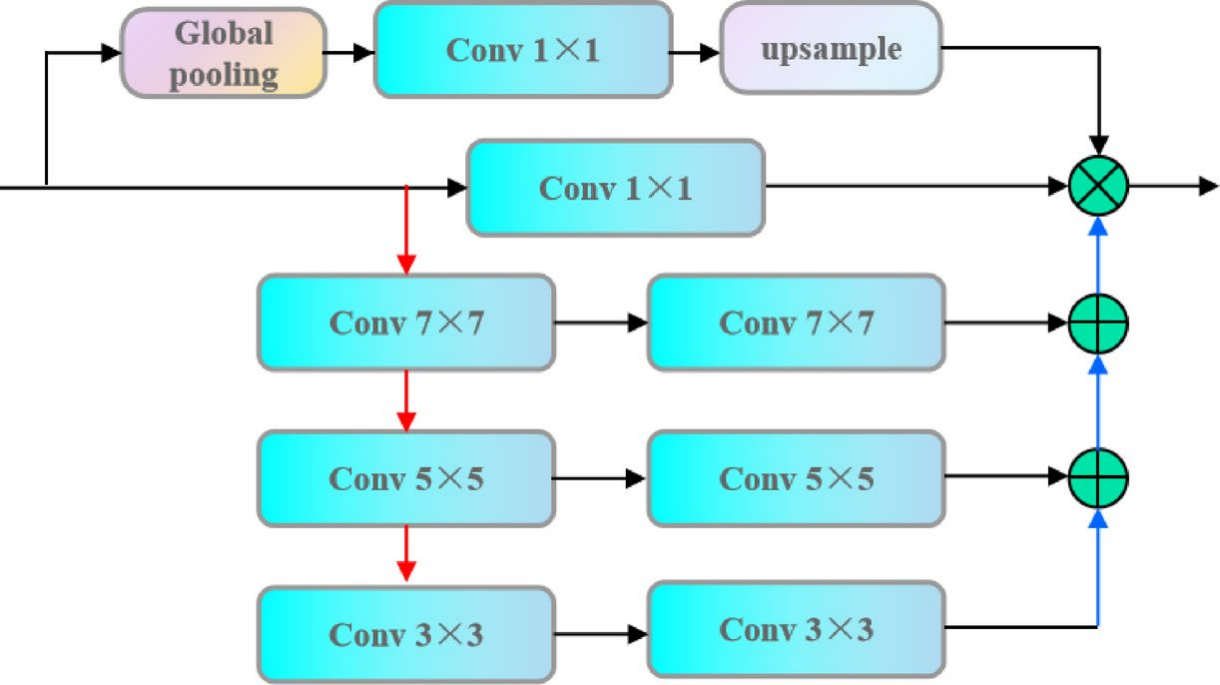
Structure of the feature pyramid.

Feature pyramid attention (FPA) mechanism^22^ enhances the feature extraction capability of the deep model, which combines the advantages of Feature Pyramid Network and the attention mechanism to better capture the feature information at different scales. The structure of Feature Pyramid Attention Network used in this paper is shown in Fig. 3.

The red lines in the figure are down-sampling operations and the blue lines are up-sampling operations. The features of different scales are fused step by step between adjacent scale layers. The fused features are multiplied with the 1 × 1 convolution kernel as well as the mask branch, respectively, to realize the extraction and fusion of features of different scales, which can effectively increase the sensory field, and its specific principle can be seen in^22^. Equation (3) is calculated as follows:

3 $$H^{\prime}(x) = \tilde{M}(x) + S^{\prime}_{3} (S^{\prime}_{2} (S^{\prime}_{1} (x))) \times \tilde{V}(x)$$

where: $x$ is the input feature data, $S^{\prime}_{1} ,S^{\prime}_{2} ,S^{\prime}_{3}$ is the parameter after the corresponding three convolutional layers in the feature pyramid attention network, $\tilde{M}(x)$ is the mask branch of the feature pyramid attention network, and $H^{\prime}(x)$ is the output feature.

## Proposed method

**Fig. 4:**
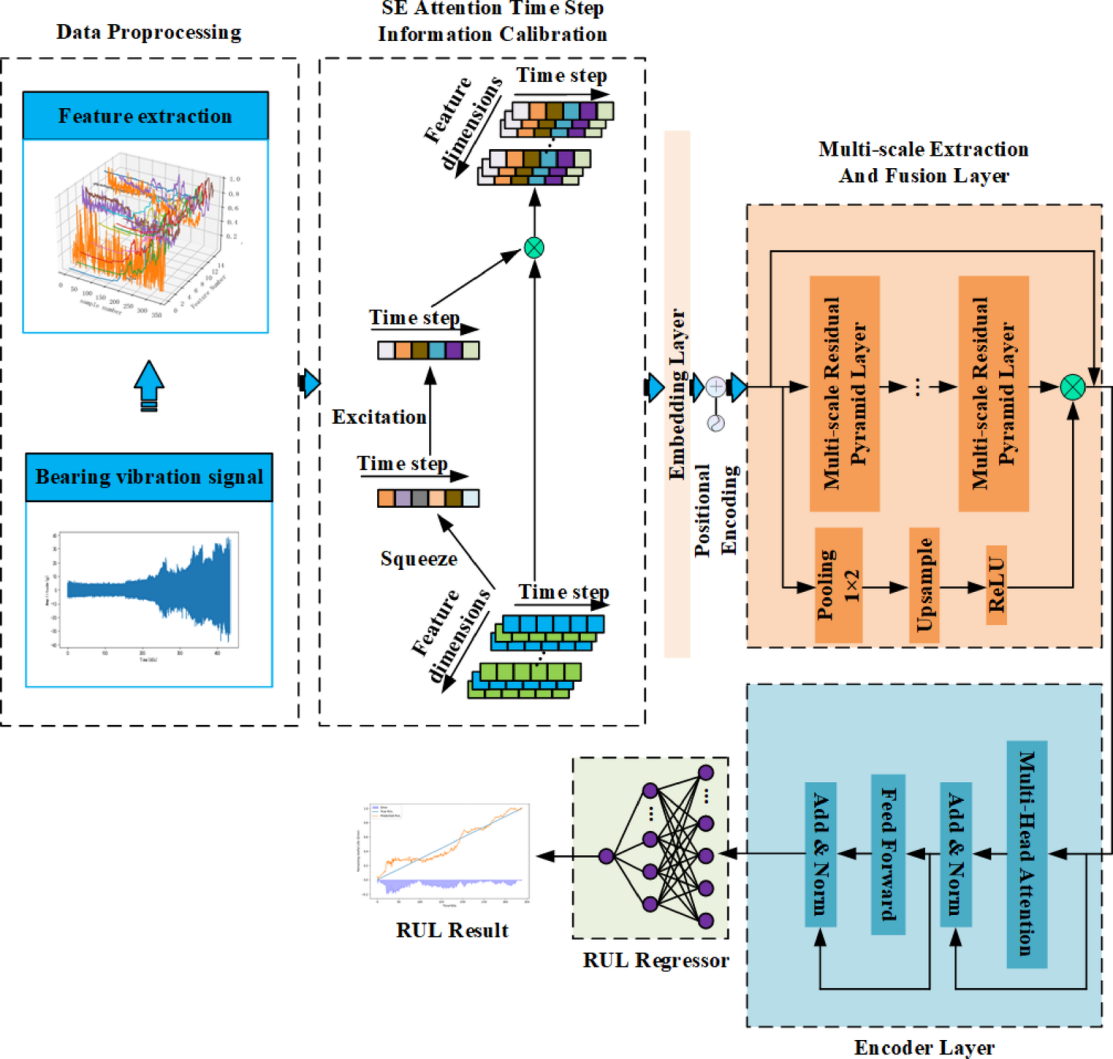
Overall life prediction process and model framework.

Aiming at the deficiencies of existing rolling bearing life prediction methods in degradation information mining, time dependency capturing, and the application of attention mechanism, this paper proposes a bearing life prediction model integrating multi-scale features and enhanced secondary attention mechanism based on the traditional Transformer structure. The model first utilizes the SE attention mechanism to calibrate the input features with time-step information and extract key state change features. Then, the multiscale feature extraction module is accessed through the embedding layer and the position encoding layer to obtain the deeply fused features. Next, the Transformer coding layer is optimized to integrate the features through the introduced multi-head attention mechanism enhanced by relative position coding. Finally, the feed-forward network captures long-term dependencies to form high-level features and outputs the predicted residual useful life (RUL) of the bearing at the regression layer. The overall framework of the model is shown in Fig. 4.

### Rolling bearing vibration state feature extraction

The huge amount of rolling bearing vibration whole-life data is prone to cause dimensional catastrophe, and the data noise will reduce the model training efficiency and increase the computational cost. The computational efficiency and prediction accuracy of the model can be effectively improved by extracting bearing statistical features.

The common time and frequency domain statistical indicators of rolling bearings are variance, mean, energy, root mean square, peak factor, crag, sum of absolute values of difference, information entropy^23^, waveform factor, impulse factor, frequency root mean square, square root magnitude, center of gravity frequency, standard deviation. The formulas are shown in Table 1.

**Table 1:** Characteristics and their corresponding formulas.

Features	Calculation formulas	Features	Calculation formulas
Variance	$X_{{\text{var}}} = \frac{1}{n}\sum\nolimits_{i = 1}^{n} {(x_{i} - \overline{X} } )^{2}$	Information entropy	$X_{se} = - \sum\limits_{i = 1}^{n} {p(x_{i} )\log_{2} [p(x_{i} )} ]$
Average value	$\overline{X} = \frac{1}{{\text{n}}}\sum\limits_{i = 1}^{n} {x_{i} }$	Waveform factor	$X_{wf} = \frac{{X_{rms} }}{{|\overline{X|} }}$
Energies	$X_{e} = \sum\limits_{i = 1}^{n} {x_{i}^{2} }$	Impulse factor	$X_{mf} = \frac{{\max (x_{i} )}}{{|\overline{X|} }}$
Root mean square	$X_{rms} = \sqrt {\frac{1}{n}\sum\limits_{i = 1}^{n} {x_{i}^{2} } }$	Root mean square of frequency	$X_{RMSF} = \sqrt {\frac{{\sum\limits_{j = 1}^{D} {f_{j}^{2} u(j)} }}{{\sum\limits_{j = 1}^{D} {u(j)} }}}$
Crest factor	$X_{cf} = \frac{{\max (|x_{i} |)}}{{X_{rms} }}$	Square root amplitude	$X_{r} = \left( {\frac{1}{n}\sum\limits_{i = 1}^{n} {\sqrt {|x_{i} |} } } \right)^{2}$
Steepness	$X_{k} = \frac{{\frac{1}{n}\sum\limits_{i = 1}^{n} {(x_{i} - \mu )^{4} } }}{{X_{{sd}}^{4} }}$	Center of gravity frequency	$X_{FC} = \frac{{\sum\limits_{j = 1}^{D} {f_{j} u(j)} }}{{\sum\limits_{j = 1}^{D} {u(j)} }}$
Sum of the absolute values of the differences	$X_{line} = \sum\limits_{i = 1}^{n} {|x_{i + 1} - x_{i} |}$	Standard deviation	$X_{sd} = \sqrt {\frac{1}{n}\sum\limits_{i = 1}^{n} {(x_{i} - \mu )}^{2} }$

In the table: $n$ represents the number of data points contained in a single sample, $x_{i}$ represents the $ith$ data point within the sample, $D$ is the total number of spectral lines, $f_{j}$ is the frequency of the $j$ spectral line, and $u(j)$ is the $jth$ spectral amplitude.

On this basis, this paper introduces the inverse tangent standard deviation and inverse hyperbolic sine standard deviation. They are obtained by transforming the data and then extracting the standard deviation from the inverse tangent (atanh) and inverse hyperbolic sine (asinh) trigonometric functions with monotonicity, respectively. Compared with the traditional features, it has better monotonicity, trend and lower scale^24^. The specific calculation formula is shown in Table 2.

**Table 2:** Standard deviation characteristics of trigonometric functions.

Features	Calculation formulas
Standard deviation of the tangent	$X_{a\tanh } = \sigma \left( {In\left[ {x_{i} + \sqrt {x_{i}^{2} + 1} } \right]} \right)$
Inverse hyperbolic sine standard deviation	$X_{a\sinh } = \sigma \left( {\frac{1}{2}In\left( {\frac{{1 + x_{i} }}{{1 - x_{i} }}} \right)} \right)$

To exclude the magnitude interference, in this paper, the acquired feature set is normalized to the maximum absolute value, and Eq. (4) is as follows:

4 $$x_{{ij}}^{\prime } = \frac{{x_{{ij}} }}{{|x_{{i\max }} |}};\quad i = 1,2,...,K;\;j = 1,2,...,N$$

where: $x_{ij}$ is the $ith$ eigenvalue of the $jth$ sample, $|x_{i\max } |$ is the absolute maximum value of the $ith$ edge column, $x^{\prime}_{ij}$ is the standardized value of the absolute value of the $ith$ feature for the $jth$ sample, $K$ the number of features, and $N$ denotes the total number of samples of the full-life data of a single rolling bearing.

### SE attention mechanism

**Fig. 5:**
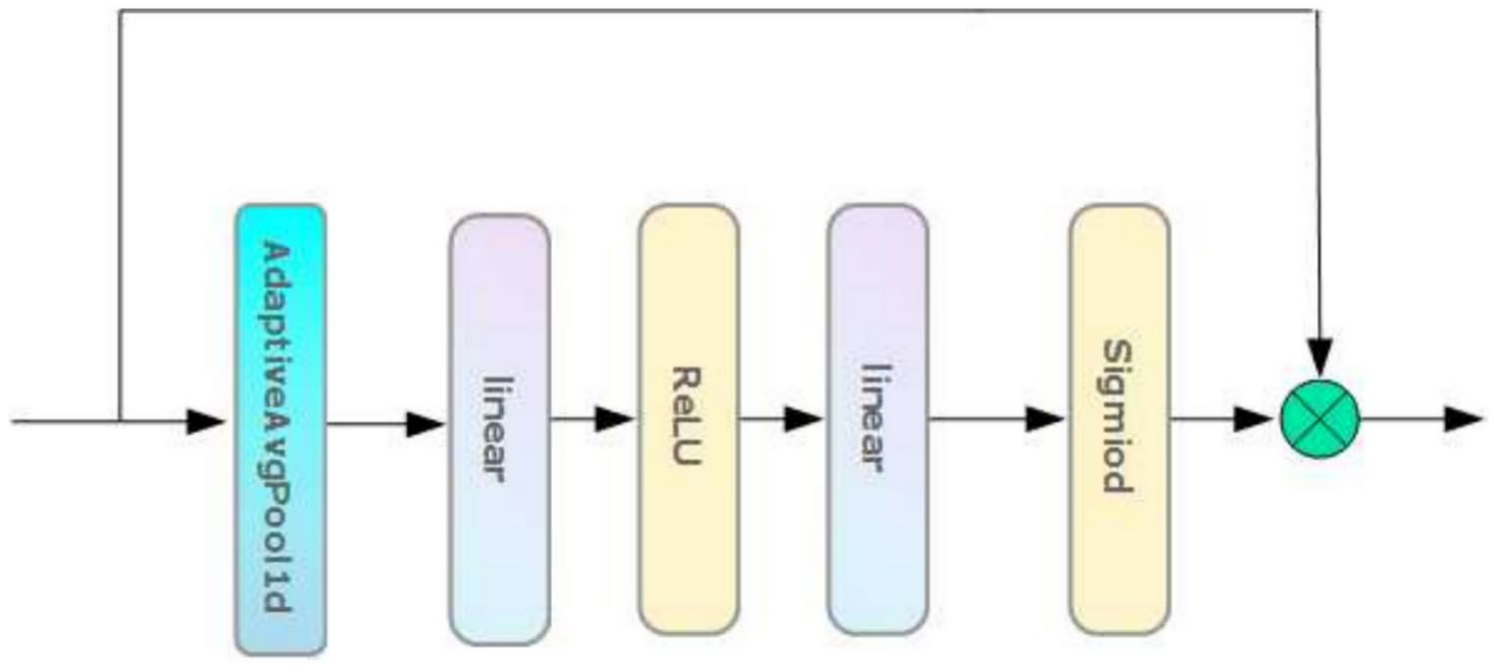
SE attention mechanism.

Squeeze-and-Excitation (SE) attention mechanism^25^ is a module for enhancing the performance of neural networks, aiming to improve the representation and generalization ability of the model by adaptively recalibrating the channel features. The SE module dynamically adjusts the weights of each channel by introducing global information to highlight the important features and improve the overall performance. The mechanism mainly consists of two parts, Squeeze and Excitation, and the specific structure is shown in Fig. 5.

The Squeeze operation compresses the global spatial information into a channel descriptor using global average pooling. Subsequently, the Excitation operation generates channel attention weights by learning and generates a re-weighted feature map with the original input data to highlight important time-step information.

In this paper, we propose to embed the time step information into the channel to calibrate the time series in the input data. This method helps the model capture the state changes of bearings at different time points and improves the model’s ability to capture bearing life characteristics. The specific Eq. (5) is as follows:

5 $$\begin{aligned} z_{c} = & F_{{sq}} \left( {u_{c} } \right) = \frac{1}{{W^{\prime } }}\sum\limits_{{i = 1}}^{{W^{\prime } }} {u_{c} (i)} \\ s = & Sigmoid\left( {W_{2} \left( {{\text{ReLU}}\left( {W_{1} z_{c} + b_{1} } \right)} \right) + b_{2} } \right) \\ \end{aligned}$$

where: $u_{c} \in R^{{B \times L \times W^{\prime}}}$ is after input time step data, $B$ is the batch size for training, $L$ is the time step length for training, $W^{\prime}$ is the length on the feature dimension of each time step i.e. 16. $z_{c} \in R^{B \times L}$ is the result of operation through Squeeze. $s$ is the result by the Excitation operation. $W_{1}$, $W_{2}$ represents trainable weights, $b_{1},$ $b_{2}$ represents trainable bias.

### Embedded layer

In the Transformer model, the main role of the Embedding Layer is to map the input data into a high-dimensional vector space to accommodate the subsequent dimensionality of the coding layer. In this case, the linear mapping is computed by Eq. (6) as follows:

6 $$y = LayerNorm(W_{l} \tilde{x} + b_{l} )$$

where: $\tilde{x}$ is the model local input data, $W_{l}$ and $b_{l}$ are the trainable weights and bias, respectively, and $y$ is the feature data normalized by layers after the output of the mapping layer.

### Positional encoding

The Transformer network obtains information about the location of features at each time step by encoding the feature data with sine and cosine functions of different frequencies between its encoding and embedding layers. It can assign a unique encoding to each position in the data sequence, assisting the model to recognize the order in the sequence. The position encoding is calculated as shown in Eq. (7).

7 $$\begin{gathered} PE(pos,2i) = \sin \left( {\frac{pos}{{10000^{{\frac{2i}{{d_{{{\text{model}}}} }}}} }}} \right) \hfill \\ PE(pos,2i + 1) = \cos \left( {\frac{pos}{{10000^{{\frac{2i}{{d_{{{\text{model}}}} }}}} }}} \right) \hfill \\ \end{gathered}$$

where: $pos$ is the feature vector position at each time step, $d_{{{\text{model}}}}$ is the coding layer dimension, $i$ belongs to any dimension in $[0,d_{{{\text{model}}}} /2)$, and $PE$ is the position embedding matrix of the feature data.

### Multi-scale residual pyramid-based feature fusion

The monitoring data of rolling bearings contain both long-term and short-term features, which will show significant differences with changes in working conditions and failure modes. Therefore, a single scale can not fully extract the local degradation information, which will lead to the deterioration of the prediction accuracy, while the feature pyramid has multi-scale characteristics, which can fully extract the global and local information, but the network becomes deeper will produce the gradient problem, and the residual attention can alleviate the gradient problem, so we combine the advantages of both of them to construct the multi-scale residual pyramid layer.

**Fig. 6:**
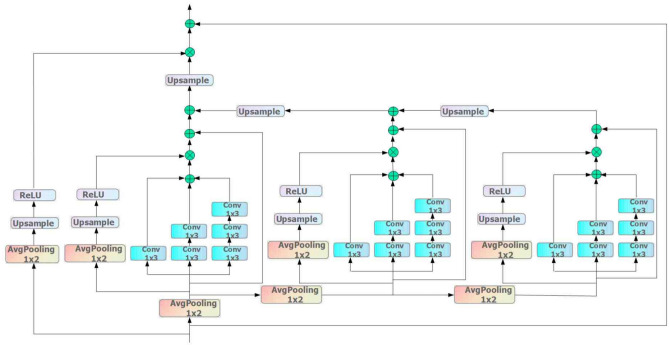
Structure of multi-scale residual pyramid layers.

As shown in Fig. 6, the multiscale residual pyramid layer used in this paper mainly consists of three identical layers of multiscale extraction blocks consisting of a combination of residual attention and feature pyramid attention, as well as the outermost residual attention mask branch for mitigating the depth of the network and residual connections. Once the features enter this layer, a downsampling operation is first performed to proportionally reduce the feature maps to 1/2, 1/4, and 1/8 sizes. Each reduced feature map then enters the multi-scale extraction module of the corresponding layer. Multiscale features are extracted in the same layer by parallel convolution operations using 1 × 3, 1 × 5, and 1 × 7 convolution kernels, respectively, and these features are additively fused. Next, feature size alignment between neighboring layers is performed by upsampling operations until fusion to the first layer. Finally, the output information is branched with the help of residual attention masks. By scaling the feature size, features of different scales are utilized in response to different receptive fields to effectively capture useful information in the data and enhance the expressive ability of the network.

Meanwhile, this paper adopts the strategy of VGGNet^26^ and Fu^27^ to use small convolutional kernels instead of large convolutional kernels, to achieve the same effect but reduce the number of parameters under the same condition of the sensory field. The specific formula (8) is as follows:

8 $$H_{i} (x) = x_{i} + S_{3} (S_{2} (S_{1} (x_{i} ))) \times M(x_{i} )$$

where: $x_{i}$ stands for the data features output from the previous network layer, $i$ represents the tandem layer number of the multiscale residual pyramid layer, $S_{1} ,S_{2} ,S_{3}$ are the network parameters of the three-layer pyramid structure, respectively, $M(x_{i} )$ is the branch of the residual attention mask, and the combination of $S_{3} (S_{2} (S_{1} (x_{i} )))$ mainly demonstrates the process of fusion between the neighboring layers of the 3 layers. $H_{i} (x)$ stands for the features that are output after passing through the multiscale residual pyramid layer.

**Fig. 7:**
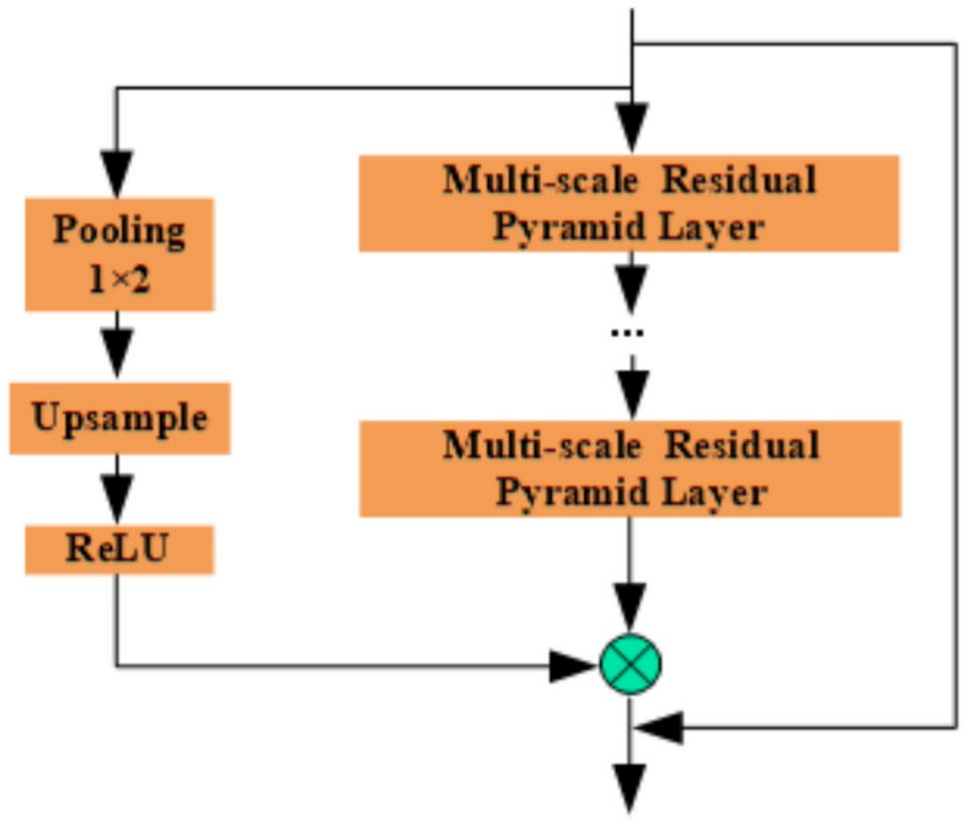
Multi-scale feature extraction fuser.

In summary, to effectively extract and fuse features of different scales and fully utilize the advantages of multiscale residual pyramid layers, this paper adopts a set of multiscale residual pyramid-based feature extraction fusion, which consists of several multiscale residual pyramid layers connected in series as well as residual attention masks and residual connections, and the structure is shown in Fig. 7. The fusionizer enhances the network’s ability to represent the data features by adding outer layers of residual attention mask branches and residual connections while avoiding the problem of disappearing or exploding gradients caused by increasing depth. At the same time, the residual attention mask branch also has the function of information correction, which helps to further enhance the feature expression ability of the network. After a large number of experiments, it is found that the multiscale residual pyramid layer 3-layer tandem is more effective, and the depth network selection and hyperparameter setting are discussed in detail in section “Deep network selection and hyper parameterization”.

### Relative positional coding for enhanced attention mechanisms

**Fig. 8:**
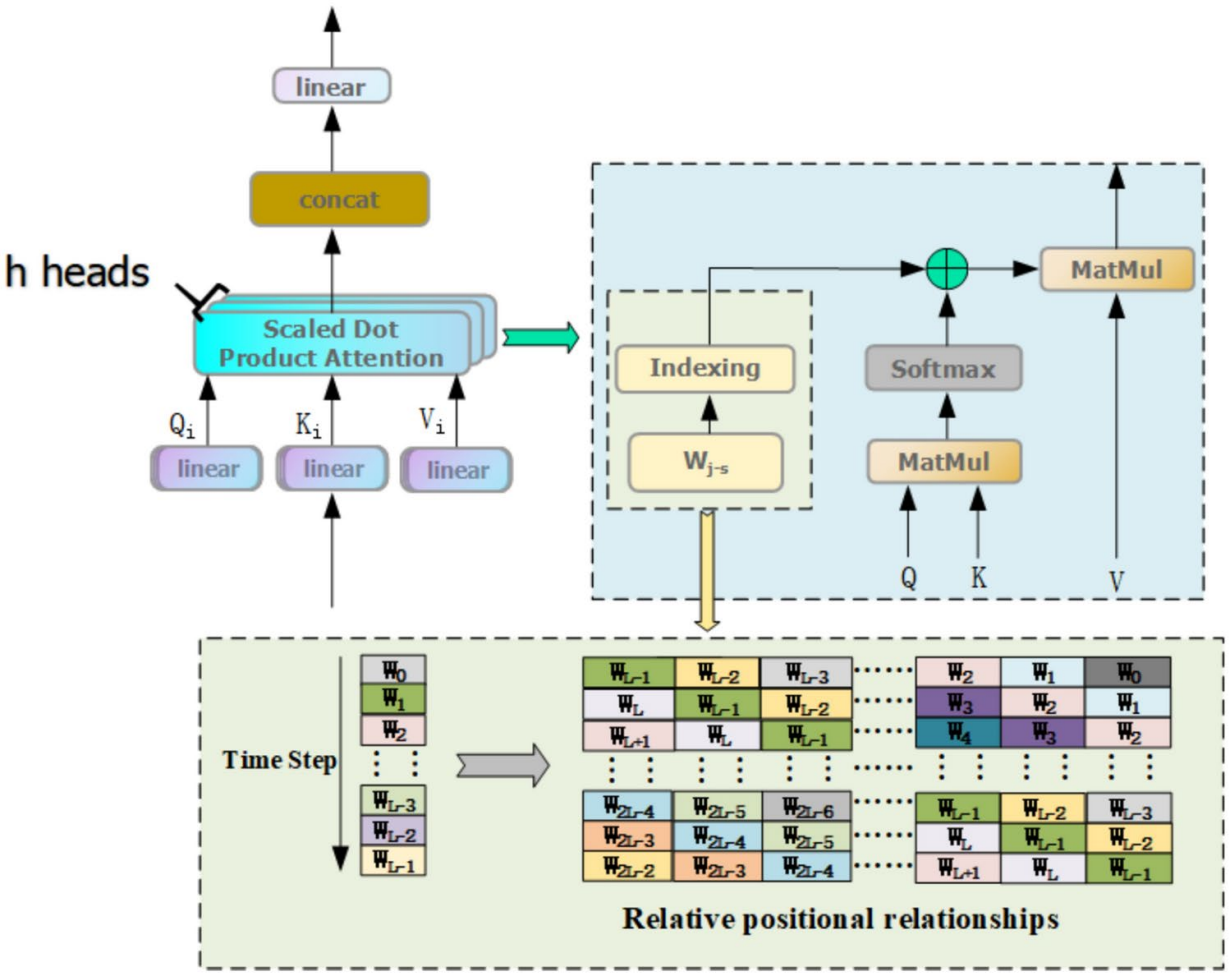
Multi-attention mechanism plus relative position-coding structure.

Rolling bearing from normal to degradation is a time-accumulation process, this paper considers from the perspective of time-step, i.e., sample location, to enhance the time-step dependency of bearing full-life characterization data in order to capture the key information. Although the Transformer models the sequence order through sine–cosine position encoding with preset frequencies, its fixed parameters are not involved in training and only provide static position characterization, which cannot model the time-step dependency. For this reason, the study introduces a relative position encoding mechanism, integrates its displacement matrix into the Softmax computation process of the multi-head self-attention mechanism, and strengthens the correlation modeling between time steps through dynamic distance weights, the specific structure of which is shown in Fig. 8.

The basic principle of relative position coding is as follows: set the coordinates (x,y) of the time step in a two-dimensional coordinate system. For simplicity, the coordinates in the x-direction are set to 0, and the coordinates in the y-direction are ordered according to the time step from 0 to the total length of the training cycle. Then, the coordinates of all training time steps (including itself) were subtracted from the coordinates of each time step to form the relative position index matrix (L × L). To ensure that the indexes were within the expected range, a constant L was added to the y-direction indexes in the matrix so that the relative position indexes for each time step were between 0 and 2L−1. The final relative position index matrix was obtained by summing the x and y direction increments in the relative position index matrix.

Next, the attention value matrix was summed with the relative position indexes, thus introducing positional information between time steps in the data, i.e., creating a trainable parameter W of size 2L−1 for each attention head, as shown in Fig. 7. These parameters reflect the maximum distance between different time steps and are also relative position weights, similar to the translation equivalence in convolution, to enhance the performance of the attention mechanism and the generalization ability of the model.

The relative position weights and attention weights are combined in Eq. (9) as follows:

9 $$a_{i} = {\text{softmax}} (X_{i} ) + W_{j - s + L - 1}$$

where: $a_{i}$ is the attention weight under the $ith$ head attention plus relative position encoding weight, $W_{j - s + L - 1}$ is the relative position weight after establishing boundary conditions between the $jth$ time step and the $sth$ time step, and $X_{i}$ is the aggregated and normalized matrix corresponding to the query matrix $Q$ and keys matrix $K$ under the $ith$ head attention.

### Feedforward layer

After the data with different weights are normalized by the layer, the data is fed into the long-range dependencies captured by the feed-forward network layer, which is formulated as follows:

10 $$FFN(x_{l} ) = \tilde{W}_{2} \cdot \max \left( {0,\left( {\tilde{W}_{1} x_{l} + \tilde{b}_{1} } \right)} \right) + \tilde{b}_{2}$$

where: $x_{l}$ is a high-level feature of the local input, $\tilde{W}_{1} ,\tilde{W}_{2}$ are trainable weight parameters, and $\tilde{b}_{1} ,\tilde{b}_{2}$ are trainable biases.

### Regressor layer

The advanced features of the feedforward network layer go through the layer normalization layer and enter the regression layer containing three linear mapping layers and two nonlinear activation functions to obtain the bearing life prediction results. The mapping is performed by reducing the dimensions layer by layer, and the dimensionality reduction in each layer can be regarded as a kind of regularization, which helps to reduce the risk of overfitting. The regression layer formula is as follows:

11 $$\mathop y\limits^{\Lambda } = W_{3} ({\text{Re}} LU(W_{2} ({\text{Re}} {\text{LU}}(W_{1} x_{t} + b_{1} ) + b_{2} )) + b_{3}$$

where: $W_{1} ,W_{2} ,W_{3}$ are the trainable parameters, $b_{1} ,b_{2} ,b_{3}$ are the corresponding trainable biases, $x_{t}$ is the high-level features of the local input, and $\mathop y\limits^{ \wedge }$ is the predicted value of the model output.

### Algorithmic flow of the prediction model

In summary, the algorithm training process of the prediction model proposed in this paper is shown in Table 3 (the overall process against Fig. 4), and the specific realization steps are as follows:

**Table 3:**
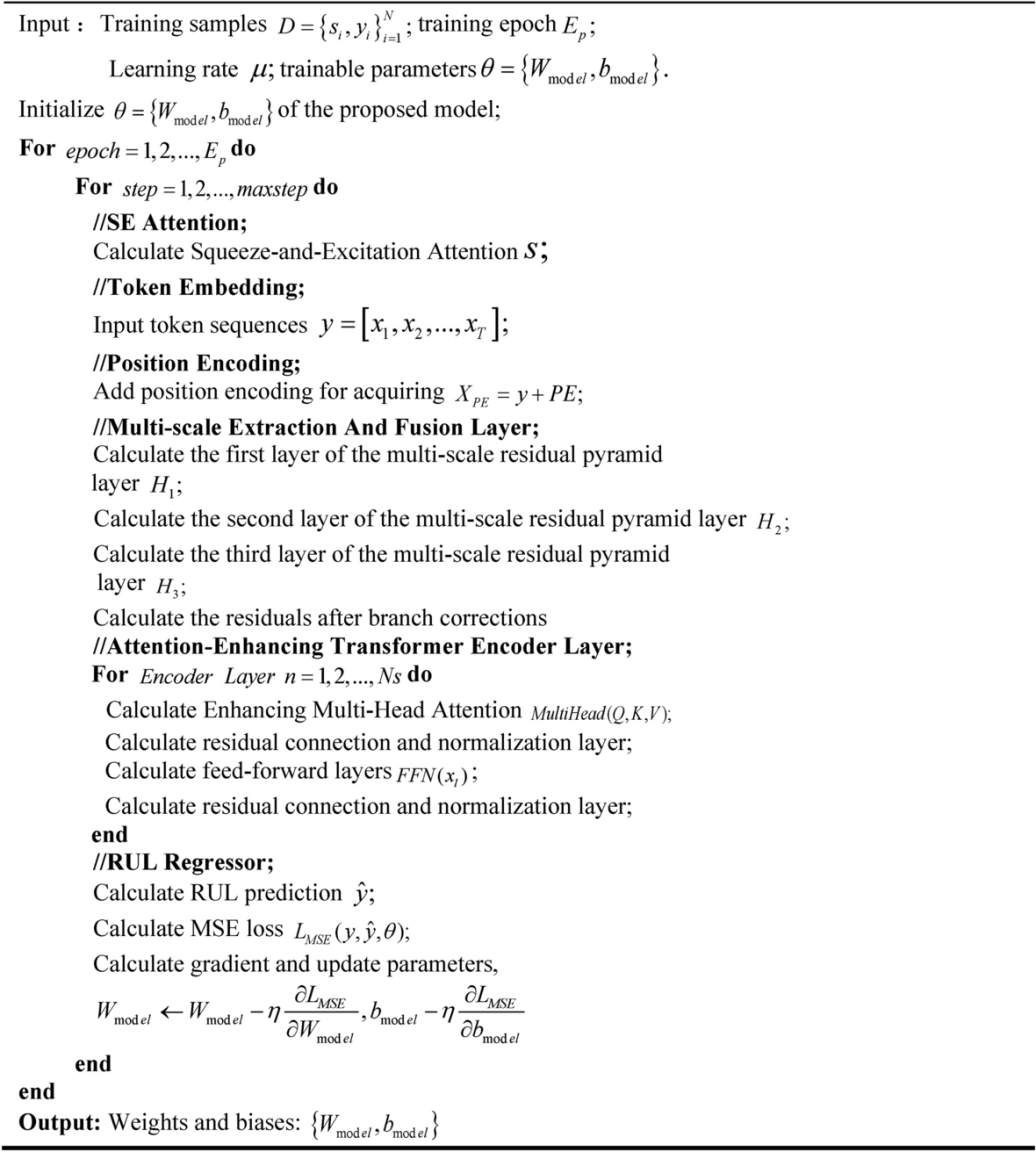
Training flow of the proposed model algorithm.

*Step 1* Feature extraction. The rolling bearing full-life public data for time domain, frequency domain and other multi-domain feature extraction, see section “Rolling bearing vibration state feature extraction ”;

*Step 2* Feature normalization and division of training and testing sets. The resulting feature set is normalized to the maximum absolute value, and the division of training and testing sets is shown in Table 5;

*Step 3* Health state labeling. The full life cycle of bearings from the divided training and test sets are normalized for use as reflecting health state labels, which are labeled within the interval from 0 to 1. The formula (12) is calculated as:

12 $$life(t) = \frac{{RUL_{t} }}{{RUL_{N} }}$$

where: $life(t)$ represents the lifetime of the bearing, and $RUL_{t}$ and $RUL_{N}$ represent the ordering t of the samples and the total number of samples in the full life cycle N, respectively.

*Step 4* Construct the SE attention module: The calibration of the bearing time step information helps the model to dynamically capture the information of the bearing state change at different time points, and then improve the model’s ability to capture the dynamic characteristics of the bearing life as well as the prediction accuracy.

*Step 5* Construct a multi-scale extraction fusionizer: A multi-scale extraction fusionizer is added between the coding and coding layers that are not involved in the training position, aiming at multi-scale extraction and fusion of the data information input into the network, and improving the richness of the key information of the network. The multi-scale extraction fusion machine realizes the degradation information mining adequately as well as the key to accurate prediction of bearing life.

*Step 6* Construct the attention-enhanced Transformer coding layer: add the relative position-coding that improves the performance of attention at the multi-head attention in the coding layer to enhance the model prediction performance and generalization.

*Step 7* Regression layer: the obtained high-level feature information characterizing the bearing life is regressed by a linear layer to get the final life prediction result.

## Experimental verification

### Data sources and evaluation indicators

The experimental data were obtained from the rolling bearing accelerated fatigue full-life dataset of Xi’an Jiaotong University^28^, which contains three working conditions, as shown in Table 4. The dataset was sampled at a frequency of 25.6 kHz, once per minute, and each sample lasted for 1.28 s.

**Table 4:** Xi’an Jiaotong University bearing acceleration fatigue experimental conditions.

Working condition	1	2	3
Speed/(r/min)	2100	2250	2400
Radial force/kN	12	11	10

Since the horizontal data of this data set contains richer information on bearing degradation^29^, after fully considering the number of samples, balance and data quality, this paper selects the horizontal data of Case 2 for method validation. The division of the specific training and testing sets is detailed in Table 5.

**Table 5:** Division of training set, test set.

Experiment	Training set	Test set
1	Bearing2_1	Bearing2_2
	Bearing2_3	
	Bearing2_5	
2	Bearing2_1	Bearing2_5
	Bearing2_2	
	Bearing2_3	

In this paper, root mean square error RMSE, mean absolute error MAE and R2 are used to evaluate the bearing life prediction effect. The lower the RMSE value, the more stable the prediction is. The lower the MAE value, the higher the accuracy of the model prediction. The closer the R2 value is to 1, the better the model prediction effect. The formula of the indicators can be found in the literature^30^.

### Deep network selection and hyper parameterization

The performance of life prediction based on deep learning depends heavily on the hyperparameter configuration. Improper selection of hyperparameters will cause the model prediction performance to deteriorate or lead to model overfitting and increased computation inside the model, which will seriously hinder the model prediction task.

In this paper, we set the training length of 40-time steps in the SE attention mechanism and set the nonlinear interaction dimension as 4 times the training length. Based on the a priori experience with the base Transformer coding layer^31^, we choose the number of coding layer layers to be 2 and the number of attention heads to be 4, considering the feature dimension of 16, the coding layer dimension is set to 64 to avoid too many parameters and overfitting. A 1–3 layer multi-scale residual pyramid structure was established respectively, and it was found that the 3-layer structure worked best, as shown in Table 6. Setting the hyperparameter range of the number of layers to 1–3 is mainly based on the following comprehensive considerations: firstly, to realize the full extraction and fusion of information through the progressive structure of the layers, and to enhance the model’s ability to capture global and local features; secondly, to construct the deep module to enhance the model’s expressive ability; and thirdly, to take into account the optimization of training efficiency while guaranteeing performance. For the initial learning rate and optimizer, the main references are the research paper set to 0.001 and the Adam optimization scheme^32^, and the learning rate decayed to 85% every 50 rounds during training. A Dropout mechanism of 0.20 is introduced in the regression layer to simulate the stochastic weight coefficient variational inference of Bayesian networks^33^. Experimental computer cloud platform testing and training CPU environment Intel(R) Xeon(R) Gold 653, GPU is NVIDIA Geforce RTX 4090, memory 24 GB, deep learning framework mainly use Pytorch version 1.7.1. The main hyperparameters of the model are detailed in Table 7.

**Table 6:** Multi-scale residual pyramid tandem layer prediction effects.

Story	Bearing2_2			Bearing2_5		
	RMSE	MAE	R2	RMSE	MAE	R2
一	0.094	0.084	0.900	0.108	0.082	0.863
二	0.074	0.065	0.941	0.182	0.146	0.625
三	0.053	0.042	0.962	0.075	0.062	0.933

**Table 7:** Main parameters of the model.

Parameter type	Parameters
Train Epoch	400
Batch size	50
Initial learning rate	0.001
Encoder layer	2
Train/test length	40
Embedding dimension	64
SE attention linear	(40,160,40)
Embedding layer(linear)	(40,64)
Multi-scale residual pyramid layer	3
Multi-attention heads	4
Optimizer	Adam
Multi-step LR ratio	0.85
Dropout rate	0.2
Regressor layer(linear)	(512,128,1)
Loss function	MSE

### Experimental results and evaluation

**Fig. 9:**
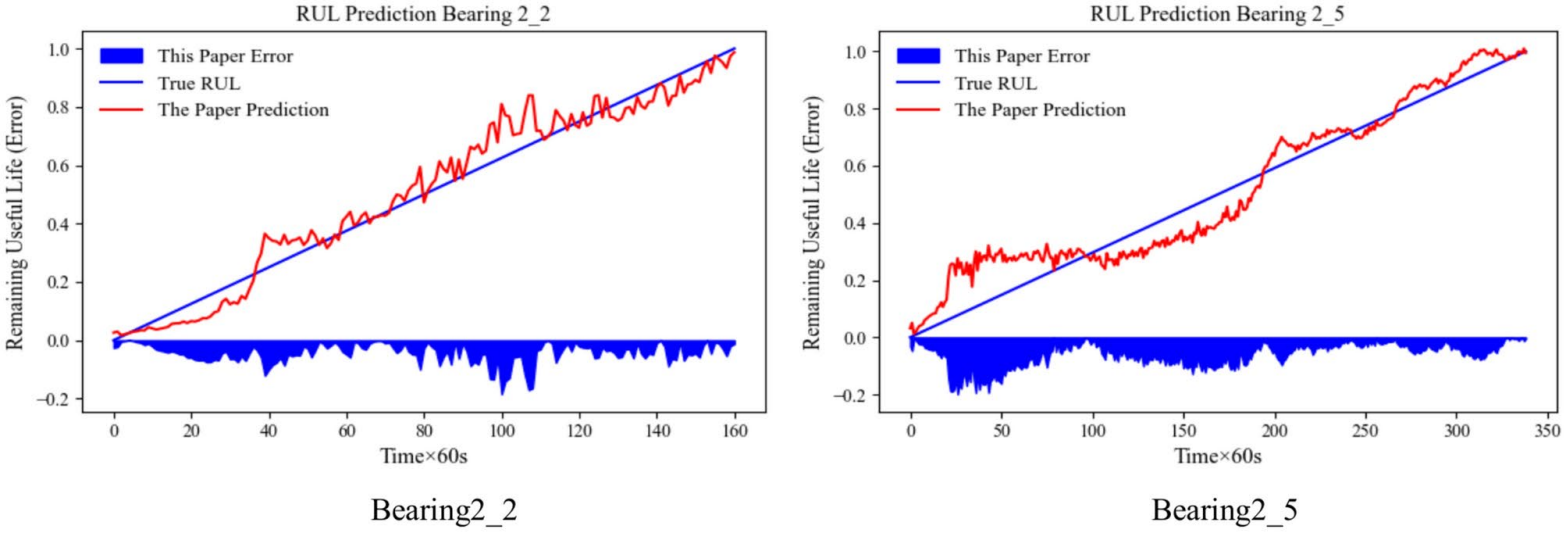
Effectiveness of model prediction.

The results of the lifetime prediction for the Bearing2_2 and Bearing2_5 data are shown in Fig. 9. The blue shaded part of the negative half-axis of the horizontal coordinate in the figure represents the absolute value deviation between the predicted RUL and the actual RUL, the red line represents the predicted RUL value, and the blue line represents the actual RUL value.

From Fig. 9, it can be seen that the two groups of experimental prediction data fluctuation are not big, can be seen in this paper proposed model has a certain robustness and stability, and at the same time observed that the later prediction is more in line with the actual life, has a higher accuracy, which is important for the later maintenance of the equipment.

The results of the proposed methods on the accuracy of rolling bearing life prediction are shown in Table 8. It can be seen that the methods proposed in this paper have high prediction accuracy.

**Table 8:** Accuracy of rolling bearing life prediction by the proposed method.

Bearing	RMSE	MAE	R2
Bearing2_2	0.05301	0.04159	0.96186
Bearing2_5	0.07505	0.06182	0.93149
Average	0.06403	0.051705	0.946675

It can be seen that the method proposed in this paper has high prediction accuracy for both sets of lifetime data.

### Algorithm comparison

**Fig. 10:**
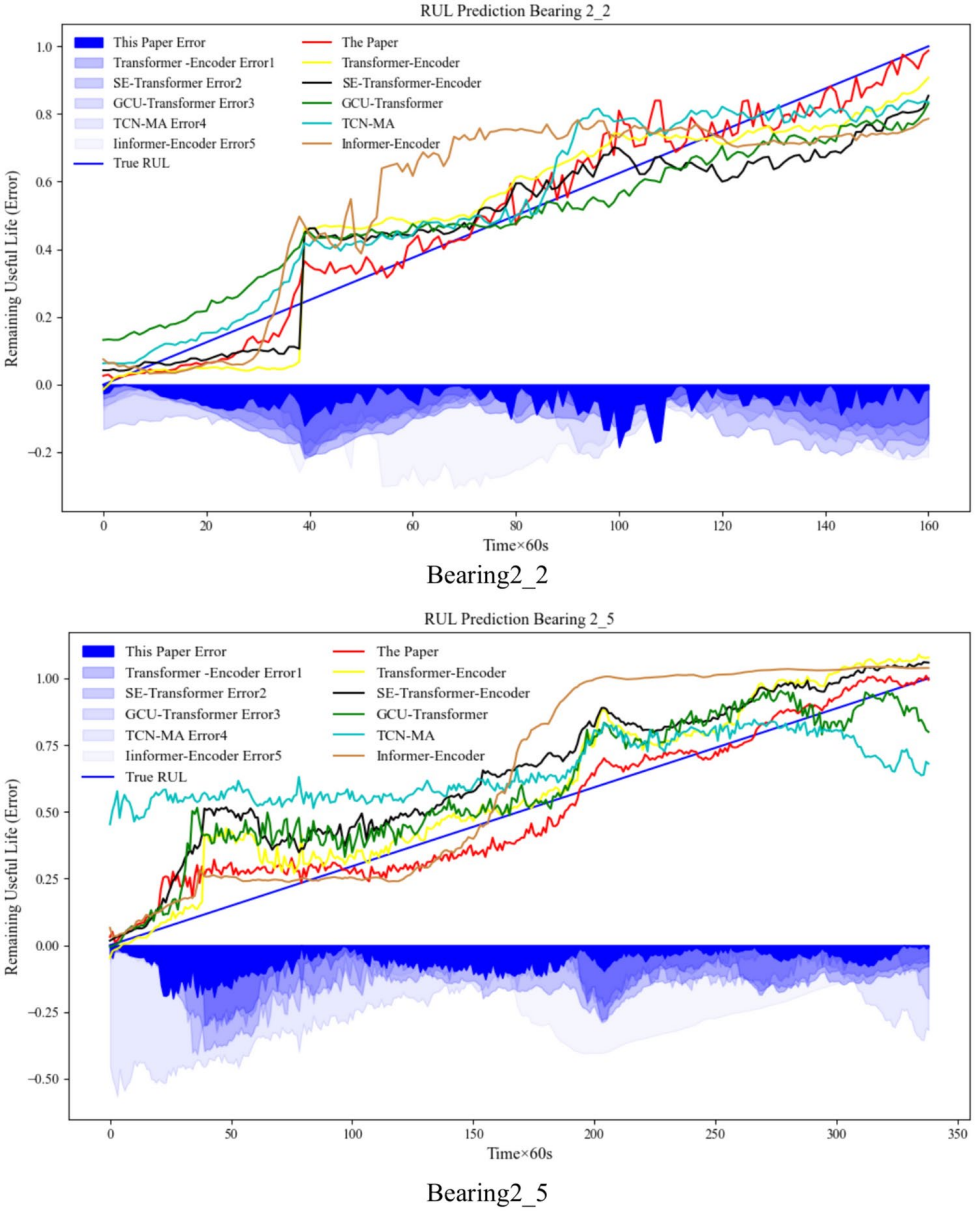
Comparison of actual RUL and model-estimated RUL and comparison method RUL.

To verify the effectiveness of the method in this paper, the life prediction models with strong relevance to the study in this paper and some emerging prediction modeling methods in recent years are selected for comparison, and the methods involved in the comparison are Transformer-Encoder, SE-Transformer-Encoder, GCU-Transformer^30^, TCN-MA^34^ and Informer-Encoder^35^. Under the conditions of hyperparameter settings that achieve the best results of each method, the comparison results are shown in Table 9 and Fig. 10.

**Table 9:** Comparison of life prediction performance of different methods.

Method	Bearing2_2			Bearing2_5		
	RMSE	MAE	R^2^	RMSE	MAE	R^2^
Transformer-encoder	0.10622	0.09614	0.87133	0.11719	0.09361	0.83622
SE-transformer-encoder	0.12456	0.10782	0.85599	0.16832	0.15123	0.65434
GCU-transformer	0.13046	0.11596	0.85614	0.13621	0.10994	0.77396
TCN-MA	0.10580	0.09077	0.89611	0.25935	0.21668	0.19472
Informer-encoder	0.17937	0.15950	0.65753	0.19575	0.15516	0.53203
This paper	**0.05301**	**0.04159**	**0.96186**	**0.07505**	**0.06182**	**0.93149**

Significance values are bold.

After comparison, it can be seen that the method of this paper has the best prediction effect, especially since the late life of the predicted value is closer to the actual life of rolling bearings. At the same time, to quantitatively observe the difference between the other methods and the method of this paper, the method of this paper (Fig. 10 red line) and the comparison method corresponding to the value of each index are subtracted, and then divided by the value of the comparison method, and then divided by the total number of indexes to obtain the error of each index is added up to the comprehensive average error E. The specific formula (13) is as follows.

13 $$\begin{gathered} E_{RMSE} = \frac{{|S_{CR} - S_{PR} |}}{{S_{CR} }} \times 100\% \hfill \\ E_{MAE} = \frac{{|S_{CM} - S_{PM} |}}{{S_{CM} }} \times 100\% \hfill \\ E_{{R^{2} }} = \frac{{|S_{CR2} - S_{PR2} |}}{{S_{CR2} }} \times 100\% \hfill \\ E = \frac{{E_{RMSE} + E_{MAE} + E_{{R^{2} }} }}{3} \times 100\% \hfill \\ \end{gathered}$$

where: $E_{RMSE}$, $E_{MAE}$ and $E_{{R^{2} }}$ represent the percentage of error of the indicator values between the comparison method and this paper’s method RMSE, MAE, and R2, respectively, and $E$ represents the combined average percentage of error of the three indicators. $S_{CR}$ represents the value of the comparison method RMSE, $S_{PR}$ represents the value of this paper’s method RMSE, $S_{CM}$ represents the value of the comparison method MAE, $S_{PM}$ represents the value of this paper’s method MAE, $S_{CR2}$ represents the value of the comparison method’s R2, and $S_{PR2}$ represents the value of this paper’s method.

Among the compared methods, the Transformer-Encoder (yellow line in Fig. 10) has global information capture and high parallel efficiency, but it is not sensitive enough to local features, which may affect the accuracy of lifetime prediction. Compared with this paper’s method, its Bearing2_2 composite average error is 39.83% higher, and Bearing2_5 composite average error is 27.10% higher, while this paper’s method has less fluctuation in the whole-life stage and more closely matches the actual life span. The SE-Transformer-Encoder (black line in Fig. 10) enhances the feature expression by front-loading SE attention but may fail to solve the problem of local feature recognition. , but may fail to solve the local feature recognition problem, resulting in 43.73% and 48.09% higher combined average errors for Bearing2_2 and Bearing2_5, respectively. GCU-Transformer (green line in Fig. 10) aims to solve the local context insensitivity problem but may be ineffective due to insufficient feature mining of the data, the Bearing2_2 and Bearing2_5 errors are 45.28% and 36.34% higher, respectively. TCN-MA (blue line in Fig. 10) combines TCN and multi-head attention, which, although it has the advantages of parallel computation and capturing long-term dependencies, is sensitive to hyper-parameters, which may affect prediction, and Bearing2_2 error is 37.14% higher. Bearing2_5 error magnitude difference is significant. Informer-Encoder (brown line in Fig. 10) improves the ability to handle long sequence data through the innovative improvement of the self-attention mechanism, but it may be due to the insufficient mining of local information and poor capturing of temporal information, which leads to the higher errors of Bearing2_2 and Bearing2_5, respectively, of 63.55% and 65.61%.

In summary, through the comparative analysis, the method in this paper considers the factors affecting the accuracy of rolling bearing life prediction more comprehensively, respectively, through the SE attention to improving the sensitivity of the prediction model to the change of the information of the data time step, at the same time by the multi-scale feature extraction fusion to deeply excavate the bearing degradation of the key information, and the use of the secondary enhancement of the attention mechanism to strengthen the link between the different time steps, which all these factors together create a good prediction performance of the proposed model. These factors together contribute to the good prediction performance of the proposed model.

## Analysis and discussion

### Ablation experiments

**Fig. 11:**
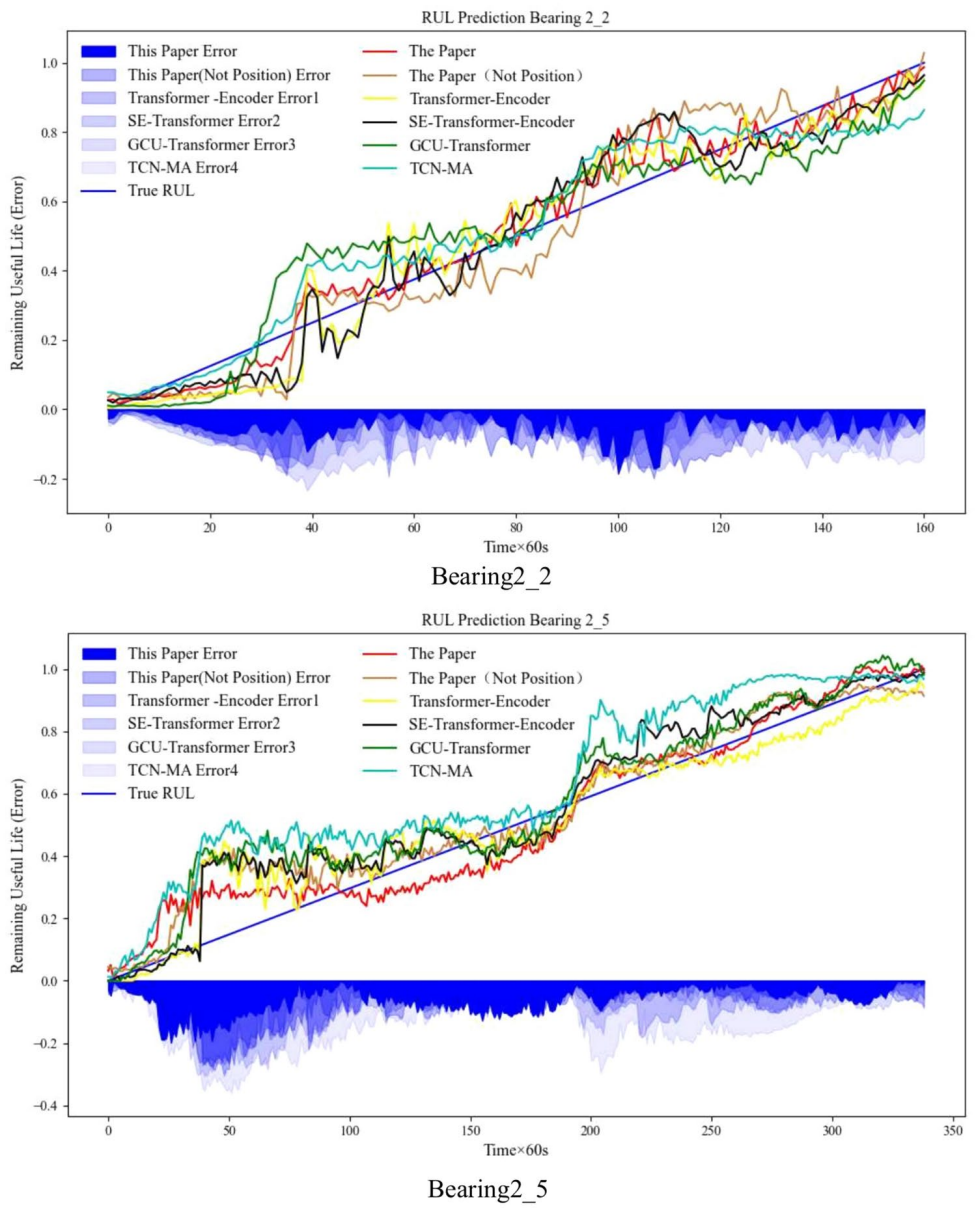
Comparison of the actual RUL and the RUL of the model estimation and comparison methods plus position coding.

To demonstrate the effectiveness of relative position coding in the proposed method, ablation experiments are carried out in this paper. Since the Informer-Encoder needs to restructure the relative position-coding embedded in sparse attention in the comparison method, this method is not included in the ablation experiments to ensure the consistency and fairness of the structure of the ablation experiments. Table 10 and Fig. 11. jointly show the effect of the ablation experiment.

**Table 10:** Adding relative position encoding model performance effect.

Method	Bearing2_2			Bearing2_5		
	RMSE	MAE	R^2^	RMSE	MAE	R^2^
Transformer-encoder	0.07556	0.06476	0.92801	0.08883	0.06501	0.90374
SE-transformer-encoder	0.07506	0.06257	0.92489	0.09520	0.07226	0.88870
GCU-transformer	0.09975	0.08664	0.87783	0.11141	0.08506	0.84853
TCN-MA	0.09868	0.08291	0.91576	0.16079	0.13466	0.68240
The paper (not position)	0.07924	0.06578	0.91421	0.08689	0.06923	0.90823
The paper	**0.05301**	**0.04159**	**0.96186**	**0.07505**	**0.06182**	**0.93149**

Significance values are bold.

Through the comparison results in Tables 9 and 10, it can be seen that this paper’s method has the best performance indexes compared with other comparison methods, and the comparison effects are shown in Figs. 10 and 11, respectively. It can be seen that the relative position coding introduced in this paper, can effectively enhance the overall model and the effectiveness of the attentional mechanism. The reason is that the relative position encoding mainly considers the relative displacement between time steps similar to the convolutional translation characteristics, which strengthens the performance of the attention mechanism and enhances the generalization of the model, and it associates the contextual information between the time steps of the time-series data, so it strengthens the model’s perception of the data. Of course, the method in this paper also has shortcomings, such as for the fusion pyramid level between the data information only through the up-sampling of the sum of the fusion, there may be information redundancy, the follow-up will be in the future to research the adaptive fusion of the model between the internal levels.

### Generalizability analysis of algorithms

To further explore the generalization of the proposed model, three different working conditions of bearing data are selected for generalization experiments.

**Fig. 12:**
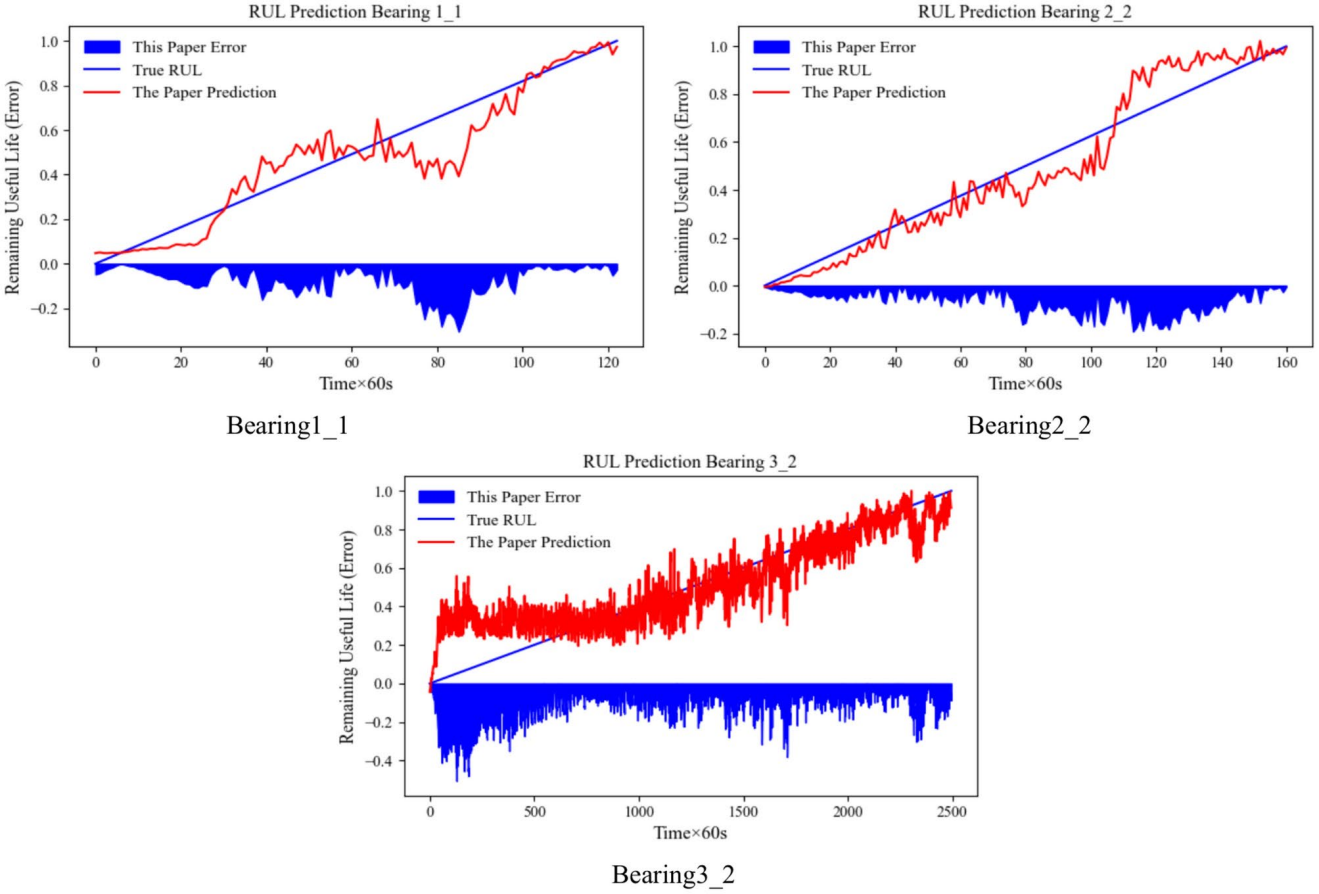
Ratio of experimental effects of actual RUL and model-estimated RUL generalization.

Firstly, bearing 1_2 under working condition 1, bearing 2_2 under working condition 2, and bearing 3_3 under working condition 3 are selected as the training set, and then bearings 1_1, 2_2, and 3_2 are selected as the test set to verify the generalization of the model. Meanwhile, to further test the superiority of the method in this paper, the comparison method in section 2.4 is added for comparison experiments. The hyperparameters are kept constant during the experiments. The results are shown in Table 11 and Fig. 12. For Bearing3_2, the experiment is carried out to the late stage for the composite fault, and it fluctuates a lot compared with the other two bearings, which may be due to the influence of the working conditions resulting in the difference in the distribution of its features caused by the RUL prediction accuracy is a little inferior. However, compared with other methods, the method in this paper still has better generalization for bearing life prediction under different working conditions, which shows the performance advantage of the method in this paper.

**Table 11:** Comparison of model methods and model generalization effect after ablation of parts.

Method	Bearing1_1			Bearing2_2			Bearing3_2		
	RMSE	MAE	R^2^	RMSE	MAE	R^2^	RMSE	MAE	R^2^
Transformer-encoder	0.13772	0.11074	0.82475	0.11468	0.09999	0.88423	0.17106	0.14684	0.64929
SE-transformer-encoder	0.11180	0.09392	0.85618	0.10314	0.08617	**0.92282**	0.16308	0.12222	0.68081
GCU-transformer	0.19874	0.16396	0.56349	0.15171	0.13223	0.75130	0.30358	0.26298	0.10523
TCN-MA	0.10643	0.07681	0.84220	0.11174	0.09597	0.85009	0.22506	0.19475	0.30798
Informer-encoder	0.17168	0.13374	0.61515	0.12318	0.10326	0.83160	0.17767	0.13940	0.62113
This paper	**0.09141**	**0.06554**	**0.88120**	**0.07636**	**0.05840**	0.91604	**0.13291**	**0.10093**	**0.78803**

Significance values are bold.

## Conclusion

This paper proposes a deep multiscale feature fusion network with dual attention for rolling bearing remaining useful life prediction, this method can effectively capture the key information in the process of rolling bearing degradation, and better improve the model’s prediction accuracy and generalization of the remaining service life of the bearing. The main research conclusions and contributions are as follows:

1. Multi-scale residual pyramid fuser architecture: a cross-layer feature extraction network is designed to synchronously capture local features and global degradation trends within the network, breaking through the limitations of single-scale modeling. Experiments show that this architecture can be used to further explore the rolling bearing degradation information.
2. Two-stage attention enhancement mechanism: A synergistic optimization scheme is proposed through SE attention (time-step dimension) and enhanced multi-head self-attention (establishment of time-step temporal dependence), which realizes the enhancement of key time-step information and the strengthening of temporal dependence between time-steps, and further improves the multi-head attention and the overall prediction performance.
3. Generalization strategy of Transformer architecture for temporal sense enhancement: Combining the previous two works, this paper propose a model of “rolling bearing remaining service life prediction based on deep network multiscale feature fusion and secondary attention mechanism enhancement”. Experiments under different working conditions show that this model can predict the remaining service life of rolling bearings more accurately than many mainstream models, and has better prediction performance.

Although the rolling bearing life prediction model proposed in this paper shows good prediction performance, there is still the problem of the model’s lightweight, which needs further attention. Future work will focus on reducing the memory footprint and complexity of the model and exploring the generalization ability of the model. In addition, further research on optimization strategies for multi-scale feature fusion will help to improve the adaptability and robustness of the model under different operating conditions.

## Data Availability

The data investigated in this paper were obtained from the publicly available dataset obtained from the full-life bearing experiments conducted by the School of Mechanical Engineering, Xi’an Jiaotong University, in conjunction with Zhejiang Changxing Shengyang Technology Co. (http://biaowang.tech/xjtu-sy-bearing-datasets).
